# Yield of MRI in patients with spontaneous deep intracerebral hemorrhage

**DOI:** 10.1007/s10140-025-02348-z

**Published:** 2025-05-20

**Authors:** Hudson McKinney, Bryan A. Kirk, Anuj J. Jailwala, Aaron McFarlane, Jackson L. Sullivan, Raghav Agarwal, Kevin D. Hiatt

**Affiliations:** Department of Radiology, Wake Forest School of Medicine, Winston-Salem, NC USA

**Keywords:** MRI, Hypertensive hemorrhage, Intracerebral hemorrhage

## Abstract

**Purpose:**

Hypertensive hemorrhage is the most common type of nontraumatic intracerebral hemorrhage (ICH), and it characteristically originates in deep structures, particularly the basal ganglia, internal capsules, thalami, brainstem, and cerebellum. While advanced imaging modalities like MRI can help uncover culprit lesions in cases of unexplained ICH, we hypothesized that the yield of brain MRI would be low in patients with spontaneous deep intracerebral hemorrhage.

**Methods:**

With IRB approval, we retrospectively reviewed cases of deep ICH at a single tertiary care academic center over a 5-year period and excluded cases with a known cause for hemorrhage. Patient history and demographics, initial blood pressure, and the results of the initial noncontrast head CT and subsequent imaging studies were recorded.

**Results:**

222 patients met study inclusion criteria, with a median age of 67 and 43.2% female sex. 188 patients (84.7%) had a history of hypertension, while 14 (6.3%) had a urine drug screen positive for cocaine or amphetamines during their hospital admission. The majority of hemorrhages were centered in the basal ganglia or internal capsules (116, 52.3%). Brain MRI was obtained for 120 (54.1%) of cases at a median interval of 0.97 days following the initial head CT, and of these studies, 85 (70.8%) included postcontrast imaging. Only 1 MRI study (0.8%) identified a culprit lesion adjacent to a cerebellar hematoma, which was later found to represent a pilocytic astrocytoma. 33.8% of patients overall met the modified Hong Kong Rule. Of the 77 MRIs performed in patients not meeting the modified Hong Kong Rule, 0 revealed a culprit lesion.

**Conclusion:**

Brain MRI obtained in the acute evaluation of patients with spontaneous deep intracerebral hemorrhage rarely uncovers a culprit lesion. Routine ordering of MRI in this cohort should be reconsidered, particularly in patients not meeting the modified Hong Kong Rule.

**Graphical Abstract:**

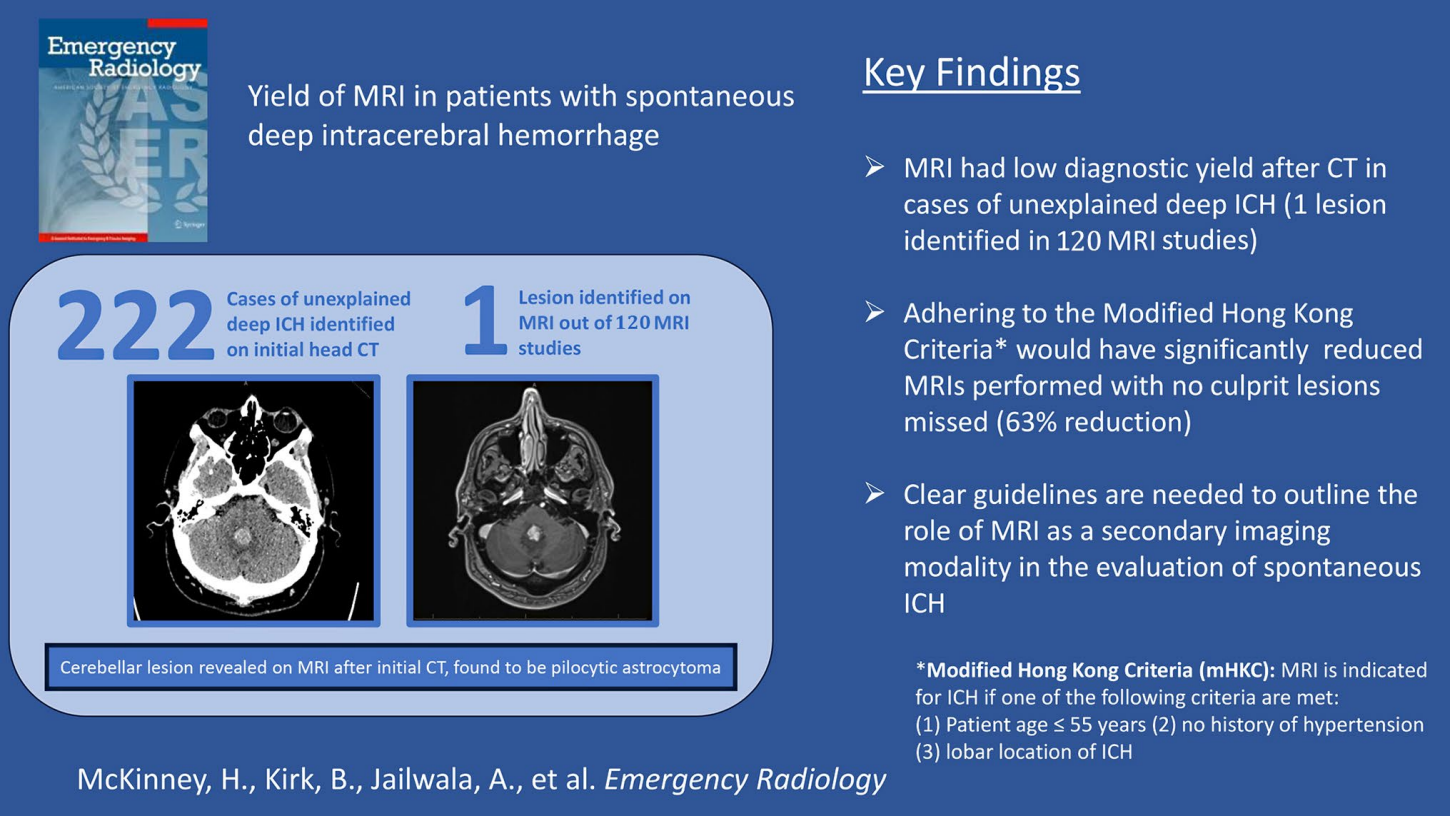

## Introduction

Spontaneous intracerebral hemorrhage (ICH) accounts for 10–15% of all strokes [1] but results in a higher proportion of stroke mortality, ranging from 40 to 60% [2]. Hypertensive hemorrhage is the most common cause of spontaneous ICH in adults, ahead of cerebral amyloid angiopathy and anticoagulation [1]. Hypertensive hemorrhage characteristically originates in deep structures, particularly the basal ganglia, internal capsules, thalami, brainstem, and cerebellum, resulting from chronic stress to the small perforating arteries and arterioles supplying these structures [3]. Chronic hypertension is also a risk factor for lobar ICH but to a lesser degree than for deep ICH [1].

Imaging is of paramount importance in the evaluation of patients with stroke-like symptoms because it helps differentiate ischemic and hemorrhagic strokes, helps identify underlying causes, and helps guide medical and surgical management. Noncontrast computed tomography (CT) of the head is typically the initial imaging modality chosen for evaluation of suspected acute stroke due to its speed, accessibility, and sensitivity for acute ICH [3]. MRI, although slower and less accessible, can offer even greater sensitivity and specificity relative to CT for detecting ICH [4]. In addition, MRI offers improved sensitivity relative to CT in uncovering culprit lesions in cases of unexplained ICH [5, 6]. However, MRI may often offer little added benefit to CT in cases where clinical history and CT findings point to a diagnosis of hypertensive hemorrhage [7].

The modified Hong Kong Rule offers a framework for determining whether to obtain MRI in patients with spontaneous ICH of unknown etiology. The original criteria based on a 1997 prospective study of patients receiving catheter angiography following ICH specify that MRI is indicated if one or more of the following criteria are met: (1) patient age less than 45 years, (2) no history of hypertension, and (3) lobar hemorrhage [8]. A 2011 study favored a modification to the criteria, increasing the age cutoff to 55 years [9].

However, the 2022 American Heart Association/American Stroke Association (AHA/ASA) Guideline for the Management of Patients with Spontaneous ICH (Sect. 4.1.2.) does not specify criteria for determining which patients should receive MRI as part of their diagnostic workup. Instead, it recommends either CT or MRI as the initial imaging modality in patients presenting with stroke-like symptoms [10]. The guideline does not favor one modality over the other but acknowledges that CT is more commonly used due to its widespread availability, rapidity, and high diagnostic accuracy and highlights the improved sensitivity added by MRI in uncovering culprit lesions [10].

To further investigate the utilization and yield of MRI in cases of spontaneous ICH, we performed a retrospective chart review at a large academic medical center. We hypothesized that the yield of brain MRI would be low in patients with spontaneous deep ICH.

## Methods

With institutional review board approval, we retrospectively reviewed cases of nontraumatic deep ICH (“deep” meaning involving the basal ganglia, internal capsules, thalami, brainstem, or cerebellum) in adult patients at a single tertiary care academic center over a 5-year period. We queried our PACS database using the Montage software (Nuance, Burlington, MA) over a 5-year period from January 1, 2018 through December 31, 2022 for “hemorrhage” or “hematoma” with any of the following locational classifiers: basal ganglia, caudate, putamen/putaminal, globus pallidus, internal capsule, thalamus/thalamic, brainstem, midbrain, pons/pontine, medulla/medullary, cerebellum/cerebellar, and vermis/vermian. Studies were excluded if there was a known cause for hemorrhage, including trauma, recent surgery, neoplasm, vascular malformation, hemorrhagic conversion of an infarct, or severe thrombocytopenia (platelet count < 50,000).

Patient demographic data, history of hypertension, blood pressure at presentation, and history and laboratory evidence of cocaine and amphetamine use were recorded. Initial head CT reports were reviewed to determine the hematoma location, volume, and presence of intraventricular extension. Reports from follow up head CTs obtained during the patient’s hospitalization were reviewed to determine whether the patient experienced hematoma expansion, which was defined as an increase in volume between baseline and follow-up CT exceeding 6 mL or 33% of the baseline volume in concordance with previous literature [11]. No reinterpretation of imaging studies was performed as part of this study; rather, data was extracted from the existing imaging reports. When hematoma measurements were not explicitly stated, one neuroradiologist with 4 years of experience performed measurements. All imaging studies were originally interpreted by fellowship trained neuroradiologists.

Patient demographics and clinical findings were compared between the groups of patients selected for MRI and the group not selected for MRI. The Chi-square test was used to compare means for nominal variables and the Mann-Whitney U-test was used to compare means for metric variables with statistical significance defined as *p* < 0.05. Statistical analyses were performed using DATAtab (Graz, Austria).

All MR studies were performed on 1.5T or 3T scanners. Imaging protocols included at a minimum the following sequences: sagittal T1-weighted, axial T2-weighted, axial diffusion-weighted and apparent diffusion coefficient, axial T2-FLAIR, and either gradient recalled echo (GRE) or susceptibility weighted imaging (SWI). When gadolinium-based intravenous contrast was administered, the protocol additionally included axial T1-weighted pre-contrast and axial and coronal T1-weighted post-contrast sequences.

## Results

Two hundred sixty-three cases of deep ICH were identified, of which 41 had ICH of known cause (29 due to trauma, 4 due to hemorrhagic conversion of an infarct, 4 due to tumors, 2 due to vascular malformations, 1 due to recent surgery, and 1 related to a platelet count < 50,000). The remaining 222 patients had a median age of 67 (interquartile range: Q1 = 56, Q3 = 76) and 43.2% female sex. 188 patients (84.7%) had a history of hypertension, while 14 (6.3%) had a urine drug screen positive for cocaine or amphetamines during their hospital admission. 30 patients (13.5%) had neither a history of hypertension nor a urine drug screen positive for cocaine or amphetamines, though 21 of these were hypertensive at initial presentation to the emergency department. Overall, 173 of the patients (77.9%) were hypertensive (mean arterial pressure > 100 mmHg) on arrival. 116 hemorrhages (52.3%) were centered in the basal ganglia or internal capsules, 51 (23.0%) in the thalami, 33 (14.9%) in the cerebellum, and 22 (9.9%) in the brainstem.

Head CTA was obtained in 161 (72.5%) of the patients and demonstrated an underlying vascular lesion in 1 patient (dural arteriovenous fistula) and a “spot sign” [12] in 17 patients (10.6%). Hematoma expansion occurred in 49 patients overall (22.1%) and in 9 of the patients with a positive “spot sign” on CTA (52.9%).

Brain MRI was obtained for 120 (54.1%) of the patients at a median interval of 0.97 days following the initial head CT, and of these studies, 85 (70.8%) included postcontrast imaging. Table 1 summarizes the patient demographics and clinical characteristics of the groups that did (MRI+) and that did not (MRI-) receive a brain MRI as part of their in-hospital workup. The MRI- group had a significantly higher percentage of female patients compared to the MRI + group (*p* = 0.047). The initial hematoma volume was significantly larger in the MRI- group (*p* = 0.004); however, after excluding the 23 MRI- and 9 MRI + patients who passed away in the first 72 h, there was no longer a significant difference in initial hematoma volume between the groups (*p* = 0.213).

**Table 1:** Patient demographics and key clinical and imaging findings. Numbers represent mean or *n* (%). P values < 0.05 are indicated by an asterisk. MAP = mean arterial pressure

	MRI obtained	MRI not obtained	*p* value
N	120	102	
Mean age (years)	66	66	0.827
Female	54 (45)	42 (41)	0.047*
History of hypertension	99 (83)	89 (87)	0.659
Mean MAP at presentation	119	122	0.591
Intraventricular extension	44 (37)	51 (50)	0.837
Mean hematoma volume (mL)	11	21	0.004*

**Fig. 1:**
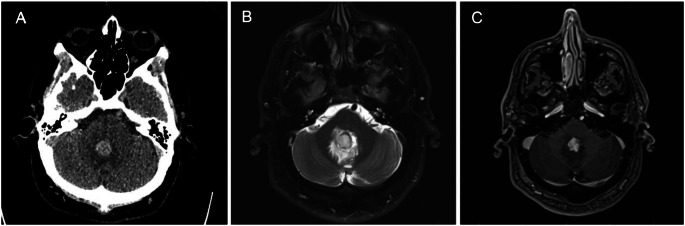
Axial noncontrast CT (**A**), T2-weighted MRI (**B**), and postcontrast T1-weighted MRI (**C**) images through the posterior fossa obtained from a 50-year-old male presenting with clinically suspected hypertensive hemorrhage. The CT image (**A**) demonstrates a round, hyperattenuating lesion in the cerebellar vermis with surrounding edema. On the subsequent MRI, the lesion demonstrates internal T2 signal hyperintensity (**B**) and solid internal enhancement (**C**)

Only 1 MRI study (0.8%) identified a culprit lesion, which revealed an enhancing mass adjacent to a cerebellar hematoma in a 50-year-old male (Fig. 1). This lesion was later confirmed to be a pilocytic astrocytoma. 75 patients overall (33.8%) and 43 of the patients who underwent MRI (35.8%) met qualifications for MRI based on the modified Hong Kong Rule. Of the 77 MRIs performed in patients not meeting the modified Hong Kong Rule, 0 revealed a culprit lesion.

## Discussion

In our retrospective review of 222 cases of unexplained spontaneous deep ICH, MRI was performed in 54.1% of cases but only identified a culprit lesion in 1 case. There remains confusion regarding the utility of MRI in the assessment of patients with spontaneous deep ICH. Current guidelines from the American Heart Association/American Stroke Association (AHA/ASA) for management of patients with spontaneous intracerebral hemorrhage do not strictly outline indications for ordering MRI versus CT for initial evaluation [10] nor for ordering MRI after an initial CT. However, the AHA/ASA guidelines do state that MRI offers the benefit of detecting both vascular malformations and possible non-macrovascular causes. In addition, the AHA/ASA guidelines point out that small vessel disease demonstrated on MRI could correlate with increased risk of ICH recurrence [10].

MRI has well-known accuracy in the diagnosis of ICH. A prospective, multicenter observational study including 62 patients with spontaneous ICH presenting within 6 h found that experienced readers achieved 100% sensitivity, specificity, predictive value, and accuracy in detecting ICH using MRI [13]. Similarly, a study of 200 patients comparing MRI and CT showed that the two modalities were equally effective in identifying acute ICH, while MRI proved more sensitive in detecting chronic ICH [4]. MRI also offers greater sensitivity compared to CT in detecting intraventricular hemorrhage (IVH) [14], which occurs in 30–50% of patients with ICH [1].

Despite the proven sensitivity and accuracy of MRI in the evaluation of patients with ICH, CT remains the first-line imaging study for the evaluation of suspected ICH at most institutions due to accessibility, cost, and speed advantages over MRI. As a result, the value added by MRI with regard to changing patient management needs to be defined to justify obtaining this additional study. While MRI certainly has the potential to uncover culprit lesions that may be occult on CT, including vascular malformations and tumors [15], an underlying lesion is not expected to be identified in cases of hypertensive hemorrhage. Therefore, it is logical to expect the yield of MRI in cases of probable hypertensive hemorrhage to be lower than in other cases of spontaneous ICH. Albeit sparse, previous literature supports a low yield of MRI in cases more likely to represent hypertensive hemorrhage. A 2015 retrospective study reviewed patients presenting with probable hypertensive ICH and found that of 222 patients with ICH, 24 received brain MRI, and only 2 of those studies (8%) revealed findings that resulted in a change in management [7]. Compared to our study, this study found a similar mean patient age of 65.6 years and similar rate of MRI utilization (50% in their study group versus 54% in ours). In a more recent review of 400 cases of MRI performed for the evaluation of spontaneous ICH, MRI revealed a culprit lesion in 12.5% of cases overall, but in 0% of cases where the patient was greater than 65 years old with a basal ganglia or thalamic hemorrhage and in 0% of cases where the patient age exceeded 85 years [16]. Similar to our study, this study found a median patient age of 65 years. The overall rate of hypertension was lower than in our study (72% versus 85%), but this study included cases of lobar and deep ICH, whereas our study only included cases of deep ICH which are more strongly associated with hypertension [1]. In support of their findings, we found a < 1% MRI yield in evaluating patients with deep ICH, with only 1 positive finding in a patient under the age of 55. Finally, although conceivable that acute blood products could obscure an underlying lesion on MRI, a retrospective study of 396 patients with spontaneous ICH demonstrated that delayed MRI was not helpful in any of the 113 patients receiving repeat MRI after negative initial MRIs [17].

With regard to the Hong Kong Rule, our study supports the modified version of these criteria proposed by Kamel et al. in which the age criterion is adjusted from < 45 years old to < 55 years old. Of the 93 MRIs performed in our study for patients over the age of 55 presenting with deep ICH of unknown etiology, 0 revealed a culprit lesion. Altogether, adhering to the modified Hong Kong Rule would have prevented 77 MRIs (64% of the MRIs performed) with no culprit lesions missed.

Beyond identifying culprit lesions, a potential benefit of MRI for ICH is providing information that helps with estimating risk for further complications. MRI findings may correlate with risk for hematoma expansion [18] as well as recurrence [19]. Imaging biomarkers found on MRI—specifically white matter hyperintensities, cerebral microhemorrhages, and cortical superficial siderosis may provide useful prognostic information. MRI can also quantify perihematomal edema, which has been shown to be predictive of hematoma expansion and need for invasive surgery [20]. However, the direct impact of these findings on patient management has yet to be determined.

This study has several limitations. Due its retrospective nature, it is difficult to determine the selection criteria used for MRI. Additionally, as this was a single-center study, the ordering patterns and imaging protocols may not match those from other institutions. Finally, most patients did not undergo delayed follow-up MRI, limiting our ability to assess long-term outcomes.

## Conclusion

We found a low yield of MRI obtained after CT in the evaluation of patients with spontaneous deep intracerebral hemorrhage. Adhering to the modified Hong Kong Rule would have allowed a 63% reduction in MRIs performed with no culprit lesions missed. While MRI is known to offer improved sensitivity over CT in the identification of causes for spontaneous ICH, its low yield for deep ICH argues against routine use for this indication.

## Data Availability

The data supporting the findings of this study are not publicly available due to sensitivity concerns but can be obtained from the corresponding author upon reasonable request.

## References

[1] Sheth KN (2022) Spontaneous intracerebral hemorrhage. N Engl J Med 387(17):1589–1596. 10.1056/NEJMra2201449 36300975 10.1056/NEJMra2201449

[2] Sacco S, Marini C, Toni D, Olivieri L, Carolei A (2009) Incidence and 10-year survival of intracerebral hemorrhage in a population-based registry. Stroke;40(2):394-9. 10.1161/strokeaha.108.523209 10.1161/STROKEAHA.108.523209 19038914

[3] Fischbein NJ, Wijman CA (2010) Nontraumatic intracranial hemorrhage. Neuroimaging Clin N Am 20(4):469–. 10.1016/j.nic.2010.07.003. 92 20974372 10.1016/j.nic.2010.07.003

[4] Kidwell CS, Chalela JA, Saver JL, Starkman S, Hill MD, Demchuk AM et al (2004) Comparison of MRI and CT for detection of acute intracerebral hemorrhage. JAMA 292(15):1823–1830. 10.1001/jama.292.15.1823 15494579 10.1001/jama.292.15.1823

[5] Moretti L, Frontera J, Lord A, Torres J, Ishida K, Czeisler B et al (2019) Performance and yield of MRI in patients with deep intracerebral hemorrhage (P3.9-001. 10.1212/WNL.92.15_supplement.P3.9-001. Neurology;92(15_supplement):P3.9-001

[6] Jain A, Malhotra A, Payabvash S (2021) Imaging of spontaneous intracerebral hemorrhage. Neuroimaging Clin N Am 31(2):193–203. 10.1016/j.nic.2021.02.003 33902874 10.1016/j.nic.2021.02.003 PMC8820948

[7] Adeli A, Behrouz R (2015) The role of magnetic resonance imaging in management of patients with nonlobar hypertensive intracerebral hemorrhage. Neurohospitalist 5(2):59–62. 10.1177/1941874414561030 25829985 10.1177/1941874414561030 PMC4357601

[8] Zhu XL, Chan MS, Poon WS (1997) Spontaneous intracranial hemorrhage: which patients need diagnostic cerebral angiography? A prospective study of 206 cases and review of the literature. Stroke;28(7):1406-9. 10.1161/01.str.28.7.1406 10.1161/01.str.28.7.1406 9227692

[9] Kamel H, Navi BB, Hemphill JC 3rd (2013) A rule to identify patients who require magnetic resonance imaging after intracerebral hemorrhage. Neurocrit Care 18(1):59–63. 10.1007/s12028-011-9607-7 10.1007/s12028-011-9607-7 21761271

[10] Greenberg SM, Ziai WC, Cordonnier C, Dowlatshahi D, Francis B, Goldstein JN et al (2022) 2022 Guideline for the Management of Patients With Spontaneous Intracerebral Hemorrhage: A Guideline From the American Heart Association/American Stroke Association. Stroke;53(7):e282-e361. 10.1161/str.0000000000000407 10.1161/STR.0000000000000407 35579034

[11] Dowlatshahi D, Demchuk AM, Flaherty ML, Ali M, Lyden PL, Smith EE (2011) Defining hematoma expansion in intracerebral hemorrhage: relationship with patient outcomes. Neurology;76(14):1238-44. 10.1212/WNL.0b013e3182143317 10.1212/WNL.0b013e3182143317 PMC3068004 21346218

[12] Wada R, Aviv RI, Fox AJ, Sahlas DJ, Gladstone DJ, Tomlinson G et al (2007) CT angiography spot sign predicts hematoma expansion in acute intracerebral hemorrhage. Stroke 38(4):1257–1262. 10.1161/01.STR.0000259633.59404.f3 17322083 10.1161/01.STR.0000259633.59404.f3

[13] Fiebach JB, Schellinger PD, Gass A, Kucinski T, Siebler M, Villringer A et al (2004) Stroke magnetic resonance imaging is accurate in hyperacute intracerebral hemorrhage: a multicenter study on the validity of stroke imaging. Stroke;35(2):502-6. 10.1161/01.Str.0000114203.75678.88 10.1161/01.STR.0000114203.75678.88 14739410

[14] Romanova AL, Nemeth AJ, Berman MD, Guth JC, Liotta EM, Naidech AM et al (2014) Magnetic resonance imaging versus computed tomography for identification and quantification of intraventricular hemorrhage. J Stroke Cerebrovasc Dis 23(8):2036–2040. 10.1016/j.jstrokecerebrovasdis.2014.03.005 25085346 10.1016/j.jstrokecerebrovasdis.2014.03.005 PMC4214254

[15] Macellari F, Paciaroni M, Agnelli G, Caso V (2014) Neuroimaging in Intracerebral Hemorrhage. Stroke;45(3):903-8. 10.1161/STROKEAHA.113.003701 10.1161/STROKEAHA.113.003701 24425128

[16] Chalouhi N, Mouchtouris N, Al Saiegh F, Das S, Sweid A, Flanders AE et al (2020) Analysis of the utility of early MRI/MRA in 400 patients with spontaneous intracerebral hemorrhage. J Neurosurg 132(6):1865–1871. 10.3171/2019.2.Jns183425 31151101 10.3171/2019.2.JNS183425

[17] Mouchtouris N, Saiegh FA, Chalouhi N, Sweid A, Papai EJ, Wong D et al (2021) Low diagnostic yield in follow-up MR imaging in patients with spontaneous intracerebral hemorrhage with a negative initial MRI. Neuroradiology;63(7):1009-12. 10.1007/s00234-020-02570-1 10.1007/s00234-020-02570-1 33226459

[18] Boulouis G, van Etten ES, Charidimou A, Auriel E, Morotti A, Pasi M et al (2016) Association of key magnetic resonance imaging markers of cerebral small vessel disease with hematoma volume and expansion in patients with Lobar and deep intracerebral hemorrhage. JAMA Neurol 73(12):1440–1447. 10.1001/jamaneurol.2016.2619 27723863 10.1001/jamaneurol.2016.2619 PMC5584595

[19] Fandler-Höfler S, Obergottsberger L, Ambler G, Eppinger S, Wünsch G, Kneihsl M et al (2023) Association of the presence and pattern of MRI markers of cerebral small vessel disease with recurrent intracerebral hemorrhage. Neurology 101(8):e794–e804. 10.1212/WNL.0000000000207510 37349111 10.1212/WNL.0000000000207510 PMC10449438

[20] Penckofer M, Kazmi KS, Thon J, Tonetti DA, Ries C, Rajagopalan S (2024) Neuro-imaging in intracerebral hemorrhage: updates and knowledge gaps. Front NeuroSci. 10.3389/fnins.2024.1408288.;18 38784090 10.3389/fnins.2024.1408288 PMC11111865

